# Supplementary material for: Inferring and validating mechanistic models of neural microcircuits based on spike-train data
Source: Nat Commun. 2019 Oct 30;10:4933. doi: 10.1038/s41467-019-12572-0 (PMC6821748; doi:10.1038/s41467-019-12572-0)
Supplement: Supplementary file 1 — Supplementary Information [file 41467_2019_12572_MOESM1_ESM.pdf]

Inferring and validating mechanistic models of neural microcircuits based on spike-train data

Ladenbauer et al.

Supplementary Methods

1. Exponential integrate-and-fire neuron model

The exponential I&F model is specified by Eqs. (5) and (6) in Methods section "I&F neuron models" with function f defined by

$$f(V) := \frac{\Delta_\mathrm{T}}{\tau_\mathrm{m}} \exp\left(\frac{V - V_\mathrm{T}}{\Delta_\mathrm{T}}\right) - \frac{V}{\tau_\mathrm{m}}, \tag{1}$$

where Δ_T is the threshold slope factor and V_T denotes the effective threshold voltage. The exponential term in Eq. (1) effectively approximates the rapidly increasing Na^+ current at spike initiation [1] and yields an improved fit to intracellular measurements of current-voltage relationships (typically obtained in-vitro) compared to the leaky I&F model [2, 3]. The value of V_s does not play an important role as long as it is sufficiently larger than the effective threshold V_T. In fact, when V increases beyond V_T, it diverges to infinity in finite time due to the exponential term, which defines a spike. In practice, however, the spike is said to occur when V reaches V_s. The exponential I&F model used here is further equipped with an absolute refractory period of length T_ref, during which $V(t)$ is clamped at V_r.

A change of V_T or Δ_T in the exponential I&F model can be completely compensated in terms of spiking dynamics by appropriate changes of μ, σ, V_r and V_s. This can be seen using the change of variables $\tilde{V} := (V - V_\mathrm{T})/\Delta_\mathrm{T}$. Consequently, the relevant parameters for inference from spike train data are μ, σ, τ_m, V_r and T_ref.

2. Numerical solution for ISI density p_ISI

Below we present two numerical solution schemes: the first one uses a finite volume discretization and is generally applicable for arbitrary variations of the input parameters, the second one uses the Fourier transform and is suitable for small amplitude variations of the parameters. Depending on the model scenario the latter scheme may be computationally more efficient than the former. Python code for both numerical schemes is available at https://github.com/neuromethods/inference-for-integrate-and-fire-models.

2.1 Solution based on finite volume discretization

This method employs a recently developed finite volume numerical scheme with implicit time discretization and Scharfetter-Gummel flux approximation, adapted from [4] for the first passage time problem. It provides an accurate solution of Eqs. (9)–(13) in Methods section "Method 1: conditioned spike time likelihood" for arbitrary variations of the mean input. In brief, we first discretize the domain $[V_\mathrm{lb}, V_\mathrm{s}]$ into N_V equidistant grid cells $[V_{m-\frac{1}{2}}, V_{m+\frac{1}{2}}]$ with centers V_m, $m \in \{1, \ldots, N_V\}$, $V_1 < V_2 < \cdots < V_{N_V}$, where $V_{\frac{1}{2}} = V_\mathrm{lb}$ and $V_{N_V+\frac{1}{2}} = V_\mathrm{s}$ are the out-most cell borders. Within each cell the numerical approximation of $p_V(V, s)$ is assumed to be constant and corresponds to the average value denoted by $p_V(V_m, s)$. Integrating Eq. (9) over the volume of cell m, and applying the divergence theorem, yields

$$\frac{\partial}{\partial s} p_V(V_m, s) = \frac{q_V(V_{m-\frac{1}{2}}, s) - q_V(V_{m+\frac{1}{2}}, s)}{\Delta V}, \tag{2}$$

where $\Delta V = V_2 - V_1$. To solve Eq. (2) forward in time (represented by the ISI variable s) the fluxes at the borders of each cell are approximated using the first order Scharfetter-Gummel flux [5],

$$q_V(V_{m+\frac{1}{2}}, s) = v_{m+\frac{1}{2}}(s)\,\frac{p_V(V_m, s) - p_V(V_{m+1}, s)\,\exp\left(-v_{m+\frac{1}{2}}(s)\Delta V/D\right)}{1 - \exp\left(-v_{m+\frac{1}{2}}(s)\Delta V/D\right)}\,, \tag{3}$$

where $v_{m+\frac{1}{2}}(s) = f(V_{m+\frac{1}{2}}) + \mu_{\mathrm{ISI}}(s)$ and $D = \frac{1}{2}\sigma^2$ denote the drift and diffusion coefficients, respectively.

For the time discretization we rewrite Eq. (2) (with Eq. (3)) in vectorized form and approximate the involved time derivative as first order backward difference to ensure numerical stability. This yields in each time step of length Δs a linear system for the vector $\mathbf{p}^{n+1}$ of probability density values at s_{n+1}, given the values $\mathbf{p}^n$ at the previous time step s_n, with vector elements $p_m^n = p_V(V_m, s_n)$,

$$(\mathbf{I} - \frac{\Delta s}{\Delta V}\,\mathbf{G}^n)\mathbf{p}^{n+1} = \mathbf{p}^n, \tag{4}$$

where $\mathbf{I}$ is the identity matrix and $\mathbf{G}^n \in \mathbb{R}^{N_V \times N_V}$ is a tridiagonal matrix that contains the discretization of the membrane voltage (cf. Eqs. (2), (3)), including the absorbing and reflecting boundary conditions (Eqs. (12) and (13) of Methods section “Method 1: conditioned spike time likelihood”).

The ISI density, Eq. (14), in this representation is obtained by evaluating the flux, Eq. (3) above, at the spike voltage V_s, taking into account the absorbing boundary condition, Eq. (12), and introducing an auxiliary ghost cell [6] with center V_{N_V+1}, which yields

$$p_{\mathrm{ISI}}(s_{n+1}|\mu_{\mathrm{ISI}}[0, s_n], \boldsymbol{\theta}) = q_V(V_{N_V+\frac{1}{2}}, s_{n+1}) = v_{N_V+\frac{1}{2}}(s_n)\,\frac{1 + \exp(-v_{N_V+\frac{1}{2}}(s_n)\Delta V/D)}{1 - \exp(-v_{N_V+\frac{1}{2}}(s_n)\Delta V/D)}\,p_{N_V}^{n+1}. \tag{5}$$

For additional details we refer to [4] (incl. supplemental material therein). Note that this method also allows for the variation of other parameters, in addition to the mean input, within the ISI. Naturally, the finite volume scheme can be used to compute the first order approximation (15) for small amplitude variations of the mean input in a straightforward way: using $J = 0$ for p_{ISI}^0, and a small value $J > 0$ to calculate p_{ISI}, to obtain $p_{\mathrm{ISI}}^1 \approx (p_{\mathrm{ISI}} - p_{\mathrm{ISI}}^0)/J$. An alternative method to compute p_{ISI}^0 and p_{ISI}^1, which may be computationally more efficient in certain cases, is presented in the next section.

2.2 Solution for weak input perturbations based on Fourier transform

The method described below is inspired by [7] and provides a solution of Eqs. (9)–(13) in Methods section “Method 1: conditioned spike time likelihood” for constant mean input, or small amplitude variations of it, through a perturbative approach. It extends the technique proposed in [7] to the case of time-varying mean input within the ISI, and can be adjusted for small amplitude variations of other parameters in a straightforward way.

We consider $\mu_{\mathrm{ISI}}(s) = \mu_{\mathrm{ISI}}^0 + J\mu_{\mathrm{ISI}}^1(s)$ with small $|J|$ according to Eq. (16). The solution of the first passage time system to first order in J can be expressed as $p_V(V, s) \approx p_V^0(V, s) + Jp_V^1(V, s)$. We insert this in the Fokker-Planck system, Eqs. (9)–(13), and neglect terms of order > 1 in J. The resulting system is then Fourier-transformed over time using separation of variables, $p_V(V, s) = x(V)y(s)$, such that $\hat{p}_V(V;\omega) = \mathcal{F}[p_V(V, s)] = x(V)\hat{y}(\omega)$, with Fourier transform and the inverse transform defined by

$$\hat{y}(\omega) := \mathcal{F}[y(s)] = \int_{-\infty}^{\infty} y(s)e^{-\mathrm{i}\omega s}ds, \qquad y(s) = \mathcal{F}^{-1}[\hat{y}(\omega)] = \int_{-\infty}^{\infty} \frac{\hat{y}(\omega)}{2\pi}e^{\mathrm{i}\omega s}d\omega, \tag{6}$$

where ω denotes angular frequency. This yields the following two (coupled) systems, one for the steady-state solution $\hat{p}_V^0(V;\omega)$,

$$\frac{d\hat{q}_V^0}{dV} = -\mathrm{i}\omega\hat{p}_V^0 \tag{7}$$

$$\frac{d\hat{p}_V^0}{dV} = \frac{2}{\sigma^2}\Big([f(V) + \mu_{\mathrm{ISI}}^0]\,\hat{p}_V^0 - \hat{q}_V^0\Big) \tag{8}$$

subject to

$$\hat{p}^0_V(V_\mathrm{s};\omega)=0, \qquad \lim_{V\to-\infty}\hat{q}^0_V(V;\omega)=0 \tag{9}$$

$$\lim_{V\searrow V_\mathrm{r}}\hat{q}^0_V(V;\omega)-\lim_{V\nearrow V_\mathrm{r}}\hat{q}^0_V(V;\omega)=1 \tag{10}$$

and one for $\hat{p}^1_V(V;\omega)$,

$$\frac{d\hat{q}^1_V}{dV}=-\mathrm{i}\omega\hat{p}^1_V \tag{11}$$

$$\frac{d\hat{p}^1_V}{dV}=\frac{2}{\sigma^2}\Big([f(V)+\mu^0_\mathrm{ISI}]\,\hat{p}^1_V+\hat{h}(V;\omega)-\hat{q}^1_V\Big) \tag{12}$$

with function $\hat{h}(V;\omega)=\mathcal{F}[\mu^1_\mathrm{ISI}(s)p^0_V(V,s)]$, subject to

$$\hat{p}^1_V(V_\mathrm{s};\omega)=0, \qquad \lim_{V\to-\infty}\hat{q}^1_V(V;\omega)=0. \tag{13}$$

The ISI density (in the frequency domain) is then given by

$$\hat{p}_\mathrm{ISI}(\omega)\approx\hat{p}^0_\mathrm{ISI}(\omega)+J\hat{p}^1_\mathrm{ISI}(\omega)=\hat{q}^0_V(V_\mathrm{s};\omega)+J\hat{q}^1_V(V_\mathrm{s};\omega). \tag{14}$$

We solve the linear ordinary differential equations (7)–(13) above for each fixed frequency ω by splitting the solutions,

$$\hat{p}^0_V(V;\omega)=\hat{p}^0_\mathrm{ISI}(\omega)\hat{p}^0_\alpha(V;\omega)+\hat{p}^0_\beta(V;\omega), \quad \hat{q}^0_V(V;\omega)=\hat{p}^0_\mathrm{ISI}(\omega)\hat{q}^0_\alpha(V;\omega)+\hat{q}^0_\beta(V;\omega) \tag{15}$$

and analogously for $\hat{p}^1_V(V;\omega)$, $\hat{q}^1_V(V;\omega)$. The components $(\hat{p}^0_\alpha,\hat{q}^0_\alpha)$ and $(\hat{p}^0_\beta,\hat{q}^0_\beta)$ respectively solve Eqs. (7)–(8) with $\hat{p}^0_\alpha=0$, $\hat{q}^0_\alpha=1$ and $\hat{p}^0_\beta=\hat{q}^0_\beta=0$ at $V=V_\mathrm{s}$ and $\lim_{V\searrow V_\mathrm{r}}\hat{q}^0_\beta-\lim_{V\nearrow V_\mathrm{r}}\hat{q}^0_\beta=1$. The components $(\hat{p}^1_\alpha,\hat{q}^1_\alpha)$ and $(\hat{p}^1_\beta,\hat{q}^1_\beta)$ solve Eqs. (11)–(12) with $\hat{p}^1_\alpha=0$, $\hat{q}^1_\alpha=1$ and $\hat{p}^1_\beta=\hat{q}^1_\beta=0$ at $V=V_\mathrm{s}$. These solution components can be conveniently computed by backward integration from V_s to a sufficiently small lower bound $V_\mathrm{lb}<V_\mathrm{r}$. We then obtain $\hat{p}^0_\mathrm{ISI}$ and $\hat{p}^1_\mathrm{ISI}$ by satisfying the reflecting boundary conditions (in (9) and (13)):

$$\hat{p}^0_\mathrm{ISI}(\omega)=-\frac{\hat{q}^0_\beta(V_\mathrm{lb};\omega)}{\hat{q}^0_\alpha(V_\mathrm{lb};\omega)}, \qquad \hat{p}^1_\mathrm{ISI}(\omega)=-\frac{\hat{q}^1_\beta(V_\mathrm{lb};\omega)}{\hat{q}^1_\alpha(V_\mathrm{lb};\omega)}. \tag{16}$$

The ISI density in the time domain is finally calculated by the inverse transform, $p_\mathrm{ISI}(s|\mu_\mathrm{ISI}[0,s],\boldsymbol{\theta})=\mathcal{F}^{-1}[\hat{p}_\mathrm{ISI}(\omega)]$ using Eqs. (14) and (16).

In practice, we first solve the steady-state system (7)–(10), evaluate the function $\hat{h}$ which appears in Eq. (12) and then solve the system for the change in ISI density due to the perturbation, (11)–(13). Note that knowledge of $\mu^1_\mathrm{ISI}(s)$ for $s\geq 0$ is required to calculate $\hat{h}$. If the input perturbations are given by delta pulses (as for the networks in Results section "Inference of synaptic coupling") the calculation of $\hat{h}$ is greatly simplified; e.g., for a pulse at $s=s_0$, $\mu^1_\mathrm{ISI}(s)=\delta(s-s_0)$ we have $\hat{h}(V;\omega)=e^{-\mathrm{i}\omega s_0}p^0_V(V,s_0)$.

3. Related I&F-based method for benchmarks

We considered the approach from [8] for comparison. This method infers the parameters of a generalized leaky I&F model with spike history dependence in response to a fluctuating background input and a time-varying stimulus by maximizing the spike train likelihood. For this purpose an approximation based on the Fokker-Planck equation is used [9]. The method is applicable to the model scenarios in Results sections "Inference of background inputs", "Inference of input perturbations" and "Inference of neuronal adaptation". We ensured equal conditions for the benchmarks in terms of model dynamics, parameters to be estimated, amount of observed data and computational resources. An efficient implementation in Matlab with integrated C/C++ code for accelerated program execution was used for the method from [8]; the code is available at http://pillowlab.princeton.edu/code_GIF.html. All tests were performed on the same personal computer.

Specifically, the model from [8] adapted for our benchmarks involves Eqs. (5) and (7) in Methods section "I&F neuron models", where the time-varying mean input $\mu(t)$ consists of three additive components: a constant baseline μ, perturbations given by the convolution of the kernel $k(t)$ with the sequence of perturbation pulses $x(t) = \sum_l \delta(t - \tilde{t}_l)$, i.e., $k * x(t) := \int_0^\infty k(\tau)x(t-\tau)d\tau$, and spike history-dependent input given by the convolution of kernel $h(t)$ with the spike train $s(t) = \sum_k \delta(t - t_k)$, i.e., $h * s(t)$. The kernels $k(t)$ and $h(t)$ were parametrically described using a particular set of basis functions (raised cosines with log scaling of the time axis; for details see [8]). To ensure equal numbers of estimated parameters for the different methods we used two basis functions for each kernel, whose weights were the parameters to be inferred. The basis functions were selected to yield, when equally weighted, a kernel shape that is similar to the shape of the respective equivalent kernel in our description. The selected basis functions and resulting kernels are shown in Supplementary Figs. 3e and 8d.

For benchmarks on inference of background inputs (cf. Results section "Inference of background inputs") we omitted the perturbation and spike history components, $k(t) = h(t) = 0$; for inference of input perturbations (cf. Results section "Inference of input perturbations") we set $h(t) = 0$ and measured detection sensitivity as for methods 1a and 2; for inference of neuronal adaptation (cf. Results section "Inference of neuronal adaptation") we set $k(t) = 0$.

4. Ground truth connection labels from in-vivo data

(Mono)synaptic connections were assumed to produce excess synchrony above baseline co-modulation in a short interval following PYR spikes. To generate estimated ground truth connection labels we adopted the approach in [10]. A CCG (0.4 ms binning) was computed for each PYR-INT pair using the evoked PYR spikes. For positive labels the peak of the CCG in the interval $[0.8, 2.8]$ ms needed to exceed that from the slowly co-modulated baseline, and it needed to be significantly larger than the largest peak in the anticausal direction (short negative lags). The lower frequency baseline was computed by convolving the observed CCG with a partially hollow Gaussian kernel, with a standard deviation of 10 ms and a hollow fraction of 60%. We estimated the probability of obtaining a spike count sc of n as observed (or higher) in the mth time lag within $[0.8, 2.8]$ ms of the CCG, given the expected, low frequency baseline rate $\lambda_\text{s}(m)$ using the Poisson distribution with a continuity correction,

$$P_\text{fast}(sc(m) \geq n|\lambda_\text{s}(m)) = 1 - \sum_{k=0}^{n-1} \frac{\lambda_\text{s}^k(m)e^{-\lambda_\text{s}(m)}}{k!} - \frac{\lambda_\text{s}^n(m)e^{-\lambda_\text{s}(m)}}{n!\,2}. \tag{17}$$

Similarly, we estimated the probability of obtaining the observed spike count n (or higher) in the mth time lag within $[0.8, 2.8]$ ms of the CCG as expected from the maximum rate λ_a across negative lags within $[-2, 0]$ ms using the Poisson distribution with a continuity correction,

$$P_\text{causal}(sc(m) \geq n|\lambda_\text{a}) = 1 - \sum_{k=0}^{n-1} \frac{\lambda_\text{a}^k e^{-\lambda_\text{a}}}{k!} - \frac{\lambda_\text{a}^n e^{-\lambda_\text{a}}}{n!\,2}. \tag{18}$$

Connections were labeled as synapses if $P_\text{fast} < 0.001$ and $P_\text{causal} < 0.0026$ for all (binned) lags in $[0.8, 2.8]$ ms, according to the rigorous criterion defined in [10].

Supplementary Figures

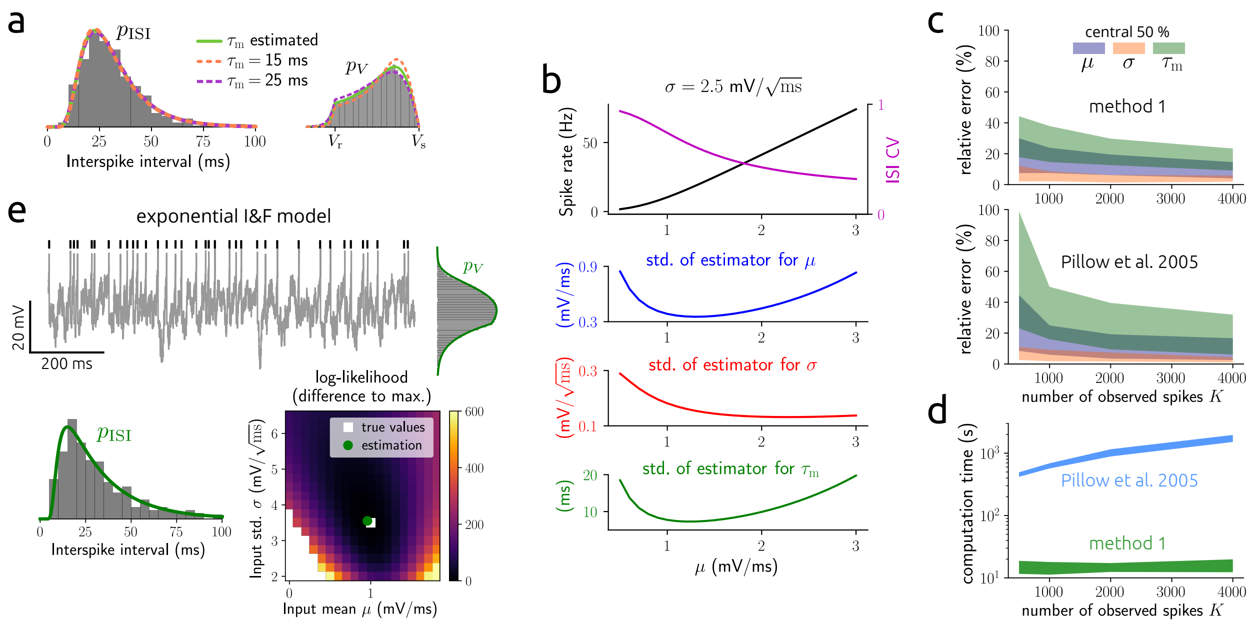

Supplementary Figure 1. Estimation and benchmark results using simulated data of single-cell spontaneous activity. a: ISI and membrane voltage histograms corresponding to the example in Fig. 1a, together with the densities p_{ISI} and p_V for τ_{m} estimated or set to wrong values (true $\tau_{\mathrm{m}} = 20$ ms). **b**, top: spike rate and ISI CV as a function of μ; below: standard deviation of estimates for μ, σ and τ_{m}, respectively, according to the Cramer-Rao bound for $K = 1000$. **c**: 25th-75th percentile (central 50 %) of relative errors between estimated and true parameter values for μ, σ and τ_{m} as a function of number of spikes; top: results from method 1, bottom: results from the method described in [8]. **d**: central 50 % of computation times corresponding to the estimations in **c** (for details see Supplementary Methods section 3). **e**: same as Fig. 1a,b for the exponential I&F model instead of the leaky I&F model. Membrane voltage histogram and p_V reflect non-refractory times. Parameter values were $V_{\mathrm{s}} = 30$ mV, $V_{\mathrm{r}} = 0$ mV, $\tau_{\mathrm{m}} = 20$ ms, $\Delta_{\mathrm{T}} = 1.5$ mV, $V_{\mathrm{T}} = 15$ mV, $\mu = 1$ mV/ms, $\sigma = 3.5$ mV/$\sqrt{\mathrm{ms}}$, $\Delta_{\mathrm{T}} = 1.5$ mV, $T_{\mathrm{ref}} = 3$ ms and $K = 400$.

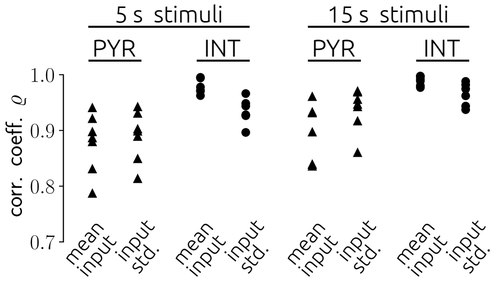

Supplementary Figure 2. Correlation between estimated input parameters and empirical input statistics from in-vitro ground truth data. Pearson correlation coefficient ϱ between estimated and empirical input statistics for all PYRs and INTs, and for two stimulus durations: 5 s-long simuli (cf. Fig. 2c) and 15 s-long stimuli (corresponding to 3 repetitions of 5 s stimuli with equal statistics).

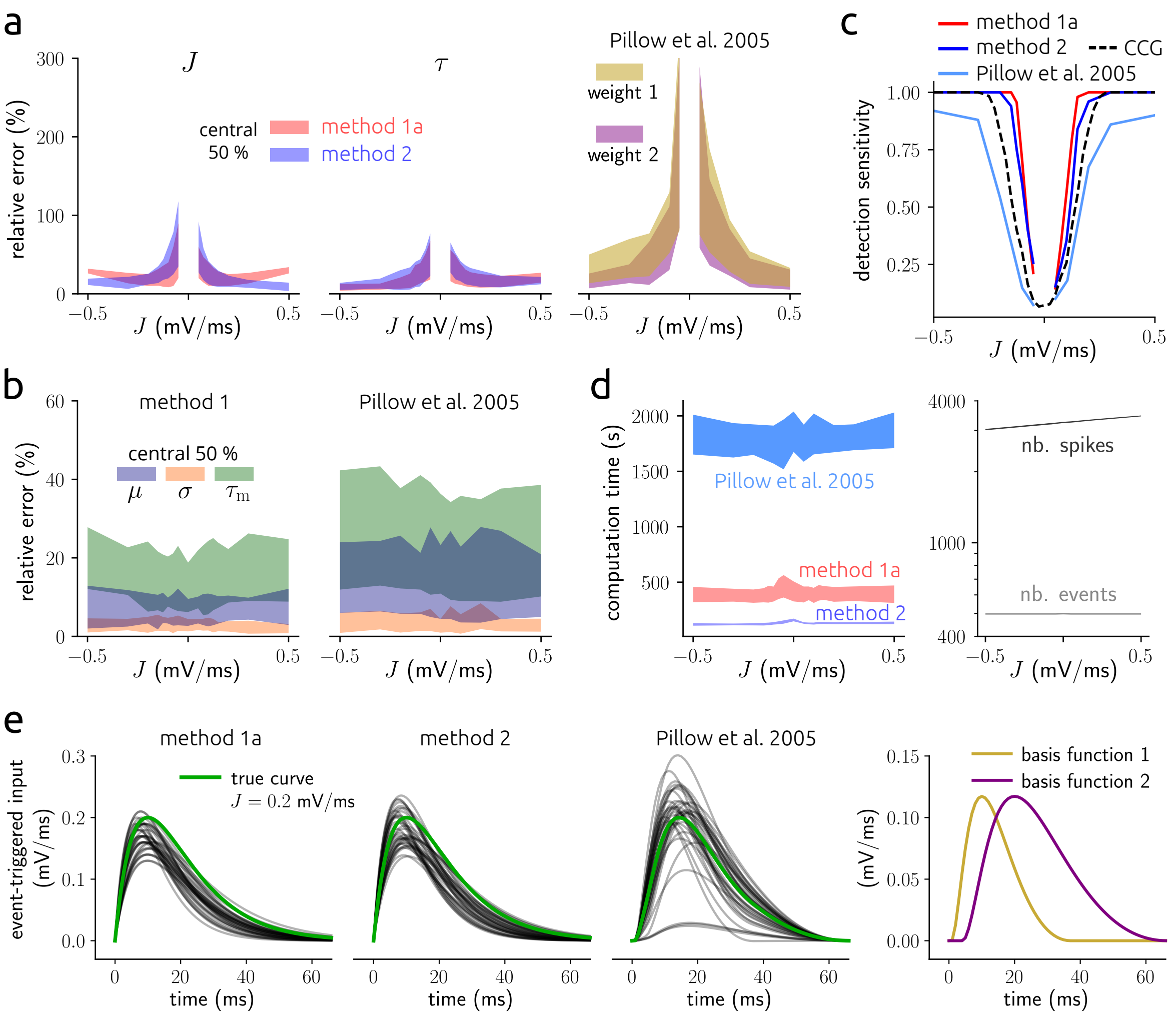

Supplementary Figure 3. Estimation and benchmark results for input perturbations using simulated data. a: 25th-75th percentile (central 50 %) of relative errors between estimated and true parameter values for J (left) and τ (center) as a function of true J using methods 1a (red) and 2 (blue), and for the two perturbation parameters using the method from [8] (right). Estimates are based on spike trains of length 100 s (for details see Supplementary Methods section 3). True $\tau = 10$ ms. **b**: central 50 % of relative errors between estimated and true parameter values for μ, σ and τ_{m} from our approach (left) and the one from [8] (right) using the same data as in **a**. Note that the parameters of input perturbations as well as μ, σ and τ_{m} were simultaneously inferred. **c**: detection sensitivity as a function of J for the methods used in **a** (solid lines) and a CCG-based method (dashed line, cf. Fig. 3d). **d**: central 50 % of computation times (left) and numbers of observed spikes and events (right) as a function of J, corresponding to the estimations in **a**–**c**. Note that slight asymmetries in **a**–**d** for excitatory compared to inhibitory perturbations may be due to differences in the number of observed spikes. **e**, left: true time series of mean input perturbation triggered at time 0 for $J = 0.2$ mV/ms (green) and reconstructions from the methods used in **a** (gray; best 75% according to the combined relative errors in **a**); right: selected basis functions for the method from [8].

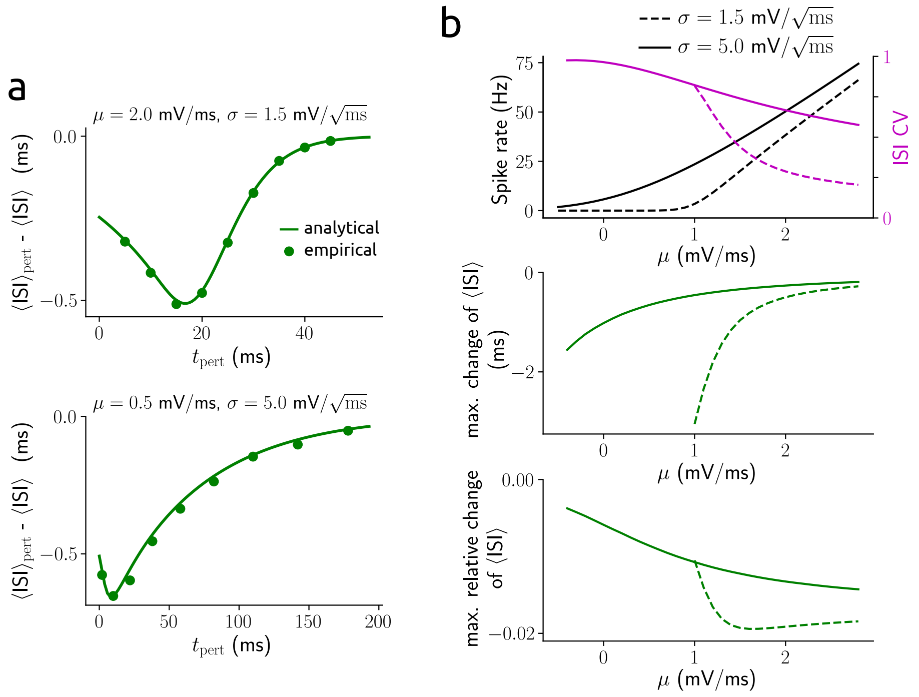

Supplementary Figure 4. Impact of weak input perturbations on the expected ISI. a: change of expected ISI due to a weak input perturbation that causes a sudden deflection of 0.5 mV in the membrane voltage ($\langle \mathrm{ISI} \rangle_{\mathrm{pert}}$ - $\langle \mathrm{ISI} \rangle$) as a function of time t_{pert} within the ISI at which the perturbation occurs, for different background input statistics. **b**, top: spike rate and ISI CV as a function of μ for different values of σ (cf. Fig. 1e); below: corresponding maximal (relative) change of expected ISI caused by an input perturbation (as in **a**).

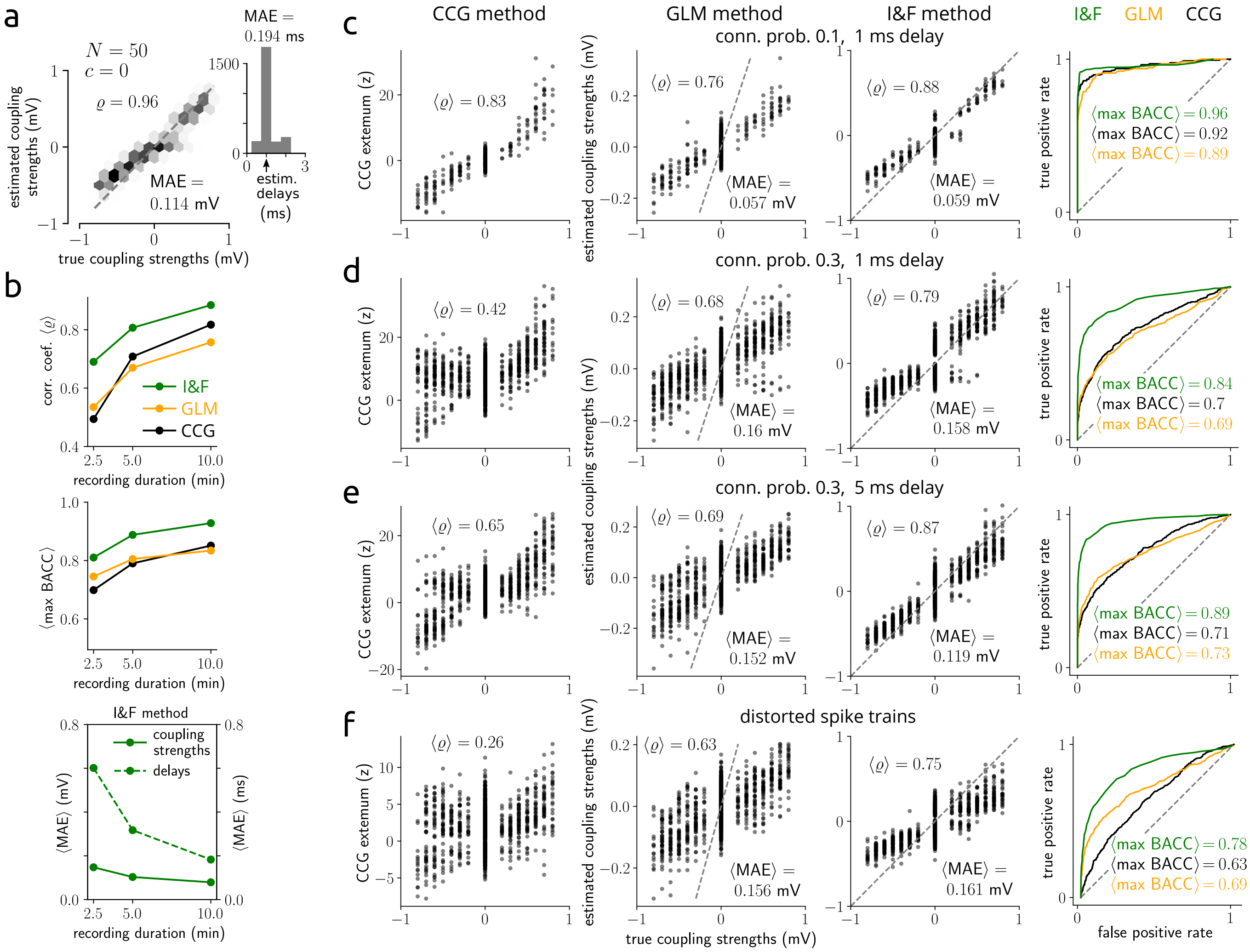

Supplementary Figure 5. Additional estimation results for synaptic coupling strengths using simulated data. a: density of estimated vs. true coupling strengths from a network of $N = 50$ neurons, with Pearson correlation coefficient ϱ and mean absolute error MAE between true and estimated coupling strengths indicated; otherwise as in Fig. 5b. **b**: average correlation coefficient $\langle\varrho\rangle$ (top) and average maximal BACC $\langle$max BACC$\rangle$ (center) across the 5 networks in Fig. 5g as a function of recording duration for the three methods (I&F, GLM, CCG); average mean absolute error $\langle$MAE$\rangle$ between true and estimated coupling strengths and delays, respectively, for the I&F method (bottom). **c**–**f**: detailed inference results from the three methods (as shown in Fig. 5d, 5e right and 5f) corresponding to the networks in Fig. 5h–k as indicated. Results from each 5 networks are shown; ROC curves represent averages.

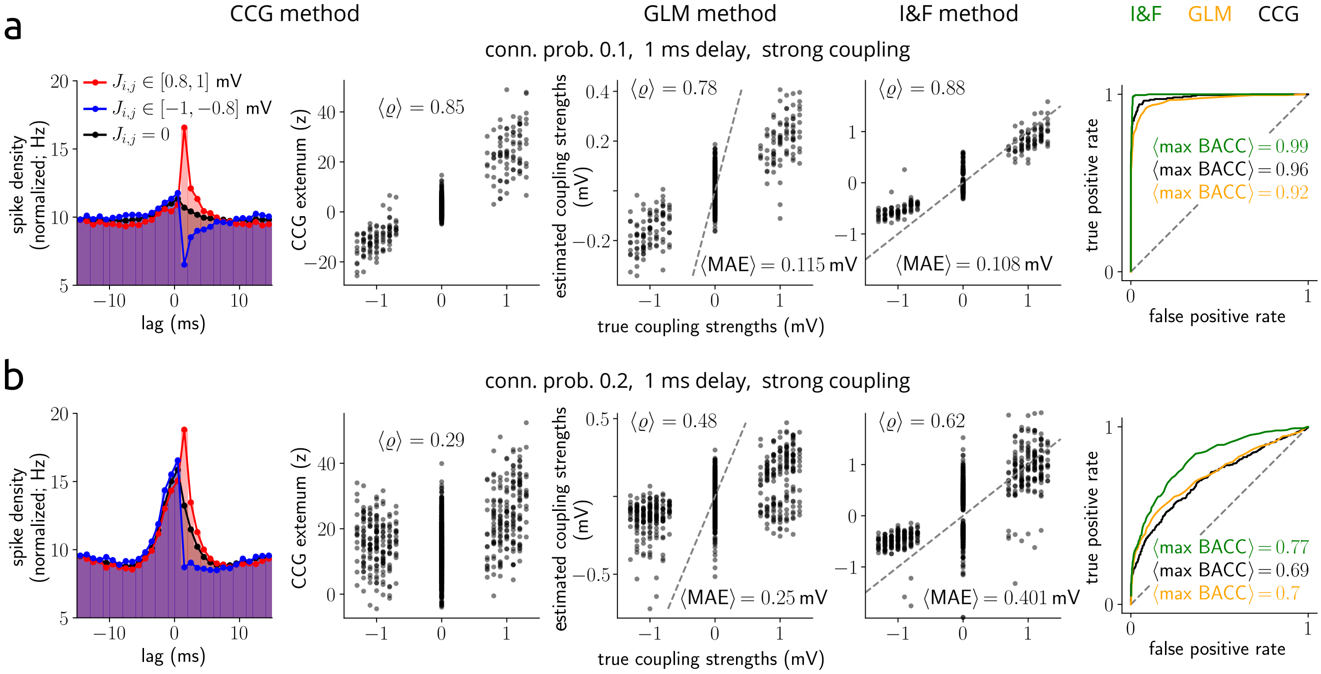

Supplementary Figure 6. Estimation results for strong synaptic coupling using simulated data. a,b: inference results from the three methods (as shown in Fig. 5d, 5e right, 5f and 5h–k left) for subsampled networks of $N_{\text{tot}} = 1000$ (500 excitatory, 500 inhibitory) neurons with connection probability of 0.1 (**a**) and 0.2 (**b**); otherwise as in Fig. 5h. Results from each 5 networks are shown; ROC curves represent averages.

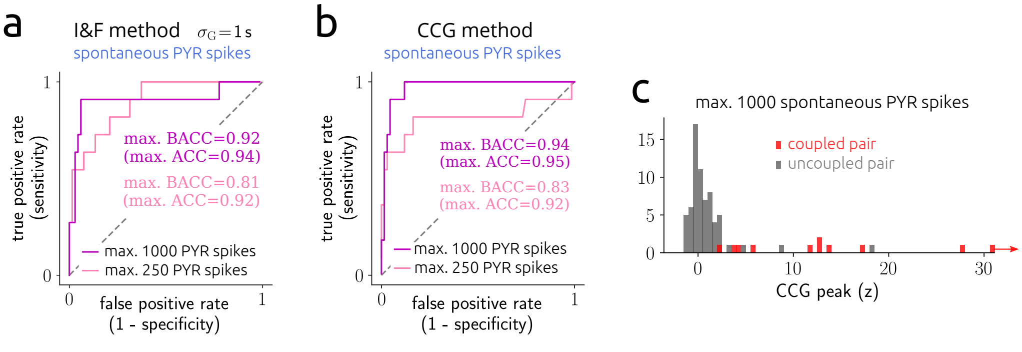

Supplementary Figure 7. Additional estimation results for synaptic couplings using in-vivo data. a: ROC curves across 78 PYR-INT pairs using the spontaneous PYR spikes for $\sigma_G = 1$ s. Maximal (balanced) accuracy values are indicated. **b**: ROC curves as in **a**, using the model-free CCG method instead of the I&F method. **c**: histogram of CCG peak z-score values for all 78 PYR-INT pairs with true labels (derived using the evoked spikes) indicated.

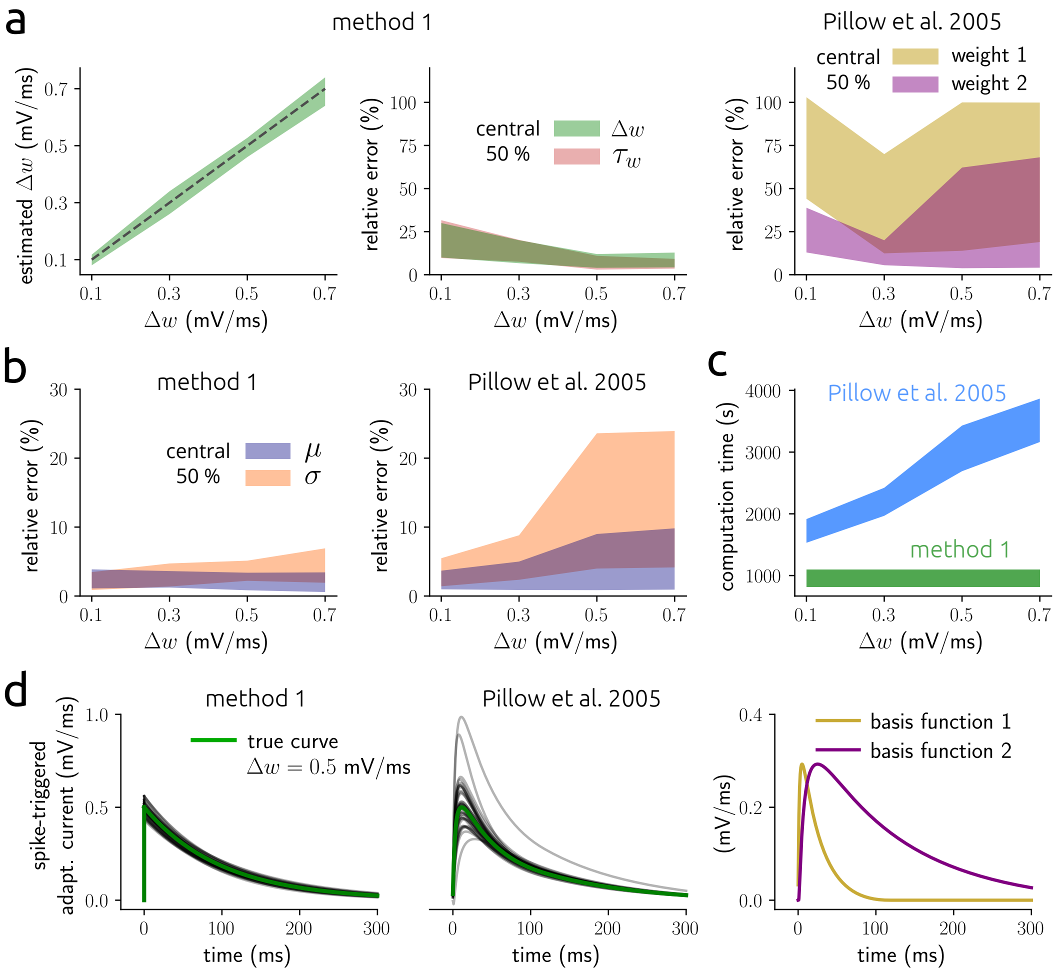

Supplementary Figure 8. Estimation and benchmark results for adaptive model neurons using simulated data. **a**, left: 25th-75th percentile (central 50 %) of estimated values for Δw as a function of true Δw using method 1; center, right: central 50 % of relative errors between estimated and true parameter values for Δw and τ_w (center), and for the two adaptation parameters using the method from [8] (right). Estimates are based on 2000 spikes (for details see Supplementary Methods section 3). True $\tau_w = 100$ ms. **b**: central 50 % of relative errors between estimated and true parameter values for μ and σ from method 1 (left) and the method from [8] (right) using the same data as in **a**. Note that the adaptation parameters as well as μ and σ were simultaneously inferred. **c**: central 50 % of computation times as a function of Δw, corresponding to the estimations in **a** and **b**. **d**, left: true time series of adaptation current triggered by a spike at time 0 for $\Delta w = 0.5$ mV/ms (green) and reconstructions from the methods used in **a** (gray; best 75% according to the combined relative errors in **a**); right: selected basis functions for the method from [8].

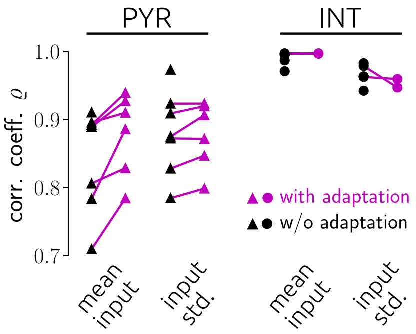

Supplementary Figure 9. Correlation between estimated input parameters and empirical input statistics for adaptive and nonadaptive models. Pearson correlation coefficient ϱ between estimated and empirical input statistics for the I&F model with adaptation (magenta symbols) and without adaptation (black symbols) for all PYRs and INTs.

Supplementary Tables

Supplementary Table 1. Computation times for estimation examples.

Figure	1a	3a†		5a‡	5c‡	7b
Method	1	1a	2	1a	1a	1
Multiprocessing	no	no	no	yes	yes	no
Estimation time (min)	< 0.2	< 10	< 2	< 17	< 117	< 7

All computations were performed on a hexa-core personal computer. †Applies to each of the two examples. ‡Computation time scaled sub-linearly with recording time (hence with number of observed spikes) and supra-linearly with number of observed neurons.

Supplementary References

[1] Fourcaud-Trocmé, N., Hansel, D., van Vreeswijk, C. & Brunel, N. How spike generation mechanisms determine the neuronal response to fluctuating inputs. *J. Neurosci.* **23**, 11628–11640 (2003).

[2] Badel, L. *et al.* Dynamic I-V curves are reliable predictors of naturalistic pyramidal-neuron voltage traces. *J. Neurophysiol.* **99**, 656–666 (2008).

[3] Harrison, P. M., Badel, L., Wall, M. J. & Richardson, M. J. E. Experimentally Verified Parameter Sets for Modelling Heterogeneous Neocortical Pyramidal-Cell Populations. *PLoS Comput. Biol.* **11**, e1004165 (2015).

[4] Augustin, M., Ladenbauer, J., Baumann, F. & Obermayer, K. Low-dimensional spike rate models derived from networks of adaptive integrate-and-fire neurons: comparison and implementation. *PLoS Comput. Biol.* **13**, e1005545 (2017).

[5] Gosse, L. *Computing Qualitatively Correct Approximations of Balance Laws*, vol. 2 (Springer, 2013).

[6] LeVeque, R. *Finite Volume Methods for Hyperbolic Problems* (Cambridge University Press, 2002).

[7] Richardson, M. J. E. Spike-train spectra and network response functions for non-linear integrate-and-fire neurons. *Biol. Cybern.* **99**, 381–392 (2008).

[8] Pillow, J. W., Paninski, L., Uzzell, V. J., Simoncelli, E. P. & Chichilnisky, E. J. Prediction and Decoding of Retinal Ganglion Cell Responses with a Probabilistic Spiking Model. *J. Neurosci.* **25**, 11003–11013 (2005).

[9] Paninski, L., Pillow, J. W. & Simoncelli, E. P. Maximum Likelihood Estimation of a Stochastic Integrate-and-Fire Neural Encoding Model. *Neural Comput.* **16**, 2533–2561 (2004).

[10] English, D. F. *et al.* Pyramidal Cell-Interneuron Circuit Architecture and Dynamics in Hippocampal Networks. *Neuron* **96**, 505–520 (2017).
