# Supplementary material for: Inferring and validating mechanistic models of neural microcircuits based on spike-train data
Source: Nat Commun. 2019 Oct 30;10:4933. doi: 10.1038/s41467-019-12572-0 (PMC6821748; doi:10.1038/s41467-019-12572-0)
Supplement: Supplementary file 3 — Reporting Summary [file 41467_2019_12572_MOESM3_ESM.pdf]

nature research

Corresponding author(s): Josef Ladenbauer
Srdjan Ostojic

Last updated by author(s): Sep 7, 2019

Reporting Summary

Nature Research wishes to improve the reproducibility of the work that we publish. This form provides structure for consistency and transparency in reporting. For further information on Nature Research policies, see Authors & Referees and the Editorial Policy Checklist .

Statistics

For all statistical analyses, confirm that the following items are present in the figure legend, table legend, main text, or Methods section.

n/a	Confirmed	
☐	☒	The exact sample size (*n*) for each experimental group/condition, given as a discrete number and unit of measurement
☐	☒	A statement on whether measurements were taken from distinct samples or whether the same sample was measured repeatedly
☐	☒	The statistical test(s) used AND whether they are one- or two-sided<br>*Only common tests should be described solely by name; describe more complex techniques in the Methods section.*
☒	☐	A description of all covariates tested
☒	☐	A description of any assumptions or corrections, such as tests of normality and adjustment for multiple comparisons
☐	☒	A full description of the statistical parameters including central tendency (e.g. means) or other basic estimates (e.g. regression coefficient) AND variation (e.g. standard deviation) or associated estimates of uncertainty (e.g. confidence intervals)
☒	☐	For null hypothesis testing, the test statistic (e.g. *F*, *t*, *r*) with confidence intervals, effect sizes, degrees of freedom and *P* value noted<br>*Give P values as exact values whenever suitable.*
☒	☐	For Bayesian analysis, information on the choice of priors and Markov chain Monte Carlo settings
☒	☐	For hierarchical and complex designs, identification of the appropriate level for tests and full reporting of outcomes
☒	☐	Estimates of effect sizes (e.g. Cohen's *d*, Pearson's *r*), indicating how they were calculated

Our web collection on statistics for biologists contains articles on many of the points above.

Software and code

Policy information about availability of computer code

Data collection	No experimental data was collected for this study.
Data analysis	Python and Matlab (MathWorks) codes were used for data analyses. Implementations of the methods used are available online, as indicated in the manuscript.

For manuscripts utilizing custom algorithms or software that are central to the research but not yet described in published literature, software must be made available to editors/reviewers. We strongly encourage code deposition in a community repository (e.g. GitHub). See the Nature Research guidelines for submitting code & software for further information.

Data

Policy information about availability of data

All manuscripts must include a data availability statement. This statement should provide the following information, where applicable:

- Accession codes, unique identifiers, or web links for publicly available datasets
- A list of figures that have associated raw data
- A description of any restrictions on data availability

No experimental data was collected for this study. The study involved available datasets from previous experimental studies with ethical approval granted. These datasets are available either online http://dx.doi.org/10.6084/m9.figshare.1144467 or from the authors on reasonable request.

Field-specific reporting

Please select the one below that is the best fit for your research. If you are not sure, read the appropriate sections before making your selection.

Life sciences study design

All studies must disclose on these points even when the disclosure is negative.

Sample size	No experimental data was collected for this study.
Data exclusions	No experimental data was collected for this study.
Replication	All results on synthetic data can be directly replicated using the available code and the information in the manuscript. Our results on experimental data can be replicated using the datasets that are available either online or from the authors. All methodological details are provided in the manuscript.
Randomization	No experimental data was collected for this study.
Blinding	Blinding was not relevant in this study.

Reporting for specific materials, systems and methods

We require information from authors about some types of materials, experimental systems and methods used in many studies. Here, indicate whether each material, system or method listed is relevant to your study. If you are not sure if a list item applies to your research, read the appropriate section before selecting a response.

Materials & experimental systems

n/a	Involved in the study	
☒	☐	Antibodies
☒	☐	Eukaryotic cell lines
☒	☐	Palaeontology
☐	☒	Animals and other organisms
☒	☐	Human research participants
☒	☐	Clinical data

Methods

n/a	Involved in the study	
☒	☐	ChIP-seq
☒	☐	Flow cytometry
☒	☐	MRI-based neuroimaging

Animals and other organisms

Policy information about studies involving animals; ARRIVE guidelines recommended for reporting animal research

Laboratory animals	No laboratory animals were used.
Wild animals	No wild animals were used.
Field-collected samples	The study did not involve samples collected from the field.
Ethics oversight	No ethical approval was required because the study did not directly involve any human or animal experiments. Our work involved available data from previous studies with ethical approval granted.

Note that full information on the approval of the study protocol must also be provided in the manuscript.
